# Association Between Perceived Stress and Post-Traumatic Stress Disorder Among Medical Staff During the COVID-19 Epidemic in Wuhan City

**DOI:** 10.3389/fpubh.2021.666460

**Published:** 2021-07-26

**Authors:** Songli Mei, Leilei Liang, Hui Ren, Yueyang Hu, Zeying Qin, Ruilin Cao, Chuanen Li, Junsong Fei, Tongshuang Yuan, Cuicui Meng, Xinmeng Guo, Jianping Lv, Yuanchao Hu

**Affiliations:** ^1^Department of Social Medicine and Health Management, School of Public Health, Jilin University, Changchun, China; ^2^The First Hospital of Jilin University, Changchun, China

**Keywords:** perceived stress, post-traumatic stress disorder, COVID-19, frontline medical staff, Insomnia severity, compassion fatigue

## Abstract

**Objective:** The study aimed to examine the relationship between perceived stress and post-traumatic stress disorder (PTSD) among frontline medical staff during the lockdown in Wuhan city, China, due to the COVID-19 outbreak.

**Methods:** The study was conducted in August 2020, which included 516 medical staff between 21 to 65 years. The PTSD Checklist-Civilian, Perceived Stress Scale, Insomnia Severity Index, and Compassion Fatigue Short Scale were used.

**Results:** The results indicated that 10.5% of the medical staff experienced PTSD symptoms, and insomnia severity mediated the effect of perceived stress on PTSD. In addition, compassion fatigue moderated the association between perceived stress and PTSD.

**Conclusion:** The study elucidated the mechanisms underlying the association between perceived stress and PTSD. Moreover, it emphasized the importance of long-term monitoring of the mental health status of frontline medical staff who supported Wuhan. The results can serve as reference for relevant medical and health departments to formulate active interventions and preventive measures against PTSD for unsung heroes who put their lives on the line during difficult times.

## Introduction

In December 2019, a new type of coronavirus (SARS-CoV-2) was reported in Wuhan, China. The World Health Organization (WHO) assigned the name COVID-19 to the resulting disease, which is characterized by acute respiratory symptoms with varying severity. COVID-19 eventually became a public health emergency at a global scale and led to tremendous impacts on public health (1). At the peak of the crisis, a large number of medical professionals in China answered the call from the government, and headed to Wuhan, the epicenter of the epidemic, to contain the situation (2). At the time, the entire city was under lockdown, and the number of patients was overwhelming. The medical staff faced many challenges, such as excessive workloads, ethical and moral conflicts, potential risk of infection in the workplace, harsh living environments, and limited medical supplies (3, 4). These factors were deemed to create high-stress and high-pressure environments, which undoubtedly led to serious psychological problems.

PTSD is a mental illness that most likely occurs in traumatized individuals during or after emergencies. It refers to continued trauma even after traumatic events, continued avoidance of stimuli related to such events, numbness, and increased arousal symptoms (5). PTSD consists of various dimensions, such as intrusion, avoidance, negative cognitive and emotional changes, and hyper-arousal (6). Previous studies found that medical staff developed varying degrees of PTSD symptoms during the COVID-19 epidemic, which indicated that they also suffered from PTSD-related symptoms (7, 8). As a result of the difficult working and living environments in Wuhan during the lockdown, the designated medical staff faced heavy stress and psychological distress, which increased the risk of developing PTSD symptoms (4). Moreover, medical staff with PTSD re-live their work experience in Wuhan through nightmares or vivid and intrusive memories, which are frequently accompanied by strong fears and physical sensations (9). These symptoms can last for at least a few weeks and can exert a serious impact on family, education, occupation, and other important life aspects of the medical staff. Thus, understanding the possible causes and influencing factors that render the medical staff assigned to Wuhan during the lockdown more vulnerable to PSTD is very important under the normalization of epidemic prevention and control.

Medical personnel, such as doctors and nurses, obtained direct contact with patients with COVID-19 due to the nature of the profession. Even with personal protective equipment, the possibility of becoming infected aroused fear among them, especially during the prophase of the epidemic where less was known about the new strain of virus. Knowing that no effective treatment or medicine exists to combat the disease created an enormous pressure on medical staff. Stress is the adaptive response of individuals to internal or external threat (10). Specifically, perceived stress refers to the degree of pressure assessed by an individual about events encountered and their ability to cope (11). During major public health emergencies, such as the COVID-19 epidemic, medical staff undergo pressure as a result of the challenges they face on a daily basis (4, 12). Previous research found that perceived stress is strongly correlated to PTSD (13).

Based on the diathesis-stress models of PTSD, traumatic events, such as the COVID-19 epidemic, were the main stimulus factors for PTSD symptoms among frontline medical staff. Moreover, the interaction with susceptibility factors is associated with the development of PTSD (14). Perceived stress was one of the psychological susceptibility factors of PTSD, such that individuals who underwent trauma were more likely to develop PTSD when faced with high levels of psychological susceptibility. Thus, this study proposes the following hypothesis:

Hypothesis 1: During the COVID-19 epidemic, the perceived stress of frontline medical staff is predicted to significantly increase PTSD.

During the early stages of the COVID-19 outbreak, insomnia was one of the main psychological conditions faced by the medical staff in Wuhan (15). Relevant research found that the prevalence of insomnia as a result of the COVID-19 epidemic among medical staff ranged from 32.0 to 49.9% (16, 17), which suggested that insomnia exerted serious impacts on physical and mental health. Adequate sleep is one of the important conditions necessary for the maintenance of physical health. Regrettably, this condition is extremely difficult to achieve for medical staff who worked in Wuhan during the lockdown. As such, they were required to deal constantly with unexpected emergencies and sudden changes in surroundings, which may lead to various sleep-related problems (18). The existing conditions, such as risk of infection, shortage of medical supplies, and inconclusive treatment plans increased the level of stress, which only aggravated sleeping problems (16, 19). Inevitably, medical staff with high levels of perceived stress are more likely to suffer from insomnia. Moreover, previous studies demonstrated that sleeping problems are closely related to PTSD (20). In fact, insomnia is one of the core symptoms of PTSD, which further indicates the correlation between insomnia severity and PTSD (6). In summary, medical staff with high levels of perceived stress are susceptible to insomnia, which can increase the chances of developing PTSD symptoms. Thus, this study proposes the following hypothesis:

Hypothesis 2: During the COVID-19 epidemic, the perceived stress of frontline medical staff can influence PTSD through the mediating effect of insomnia severity.

**Figure 1:**
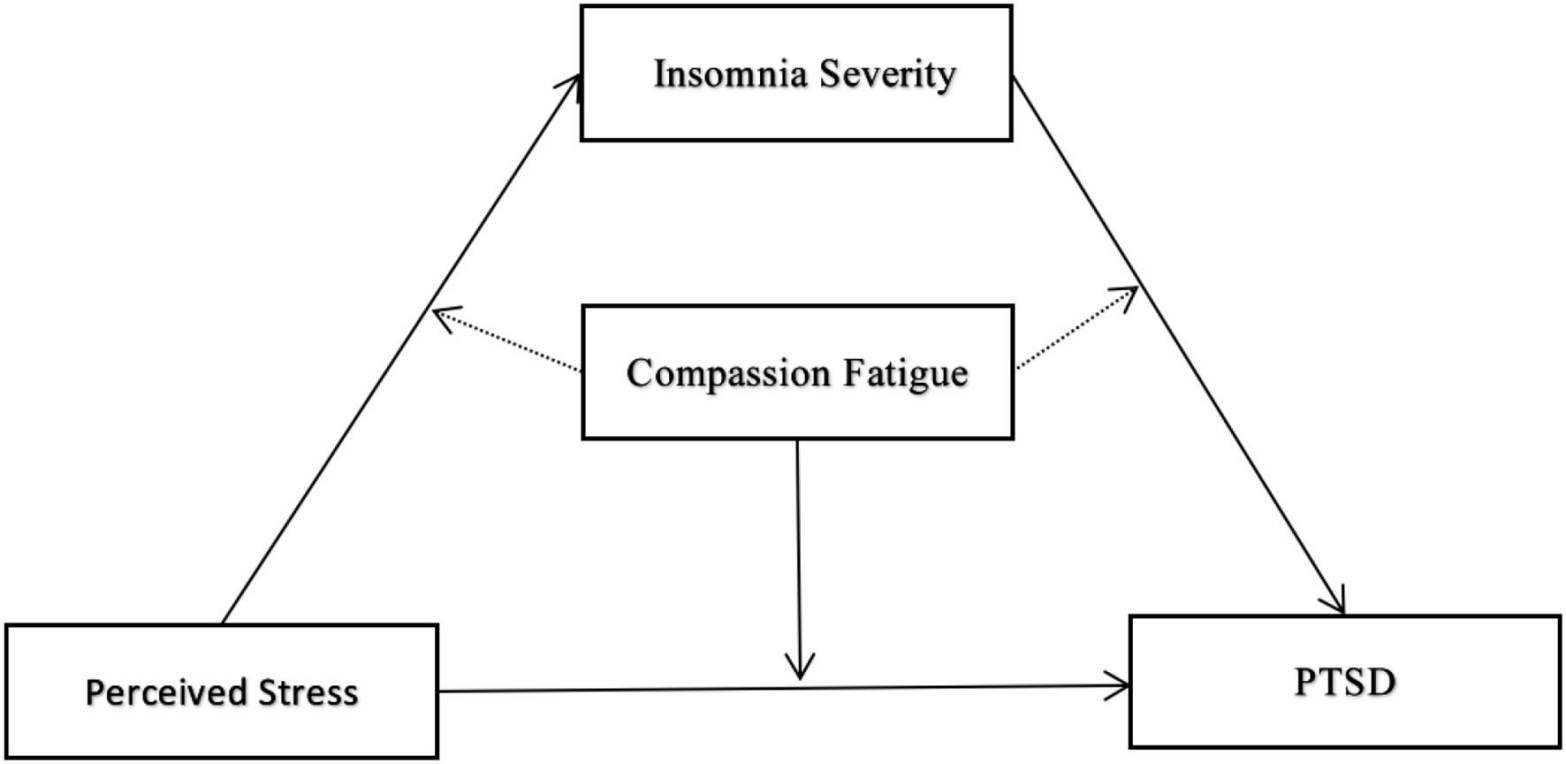
The conceptional framework of the moderated mediation model.

Compassion fatigue is an important factor related to the work pressure of medical staff (21), which is defined as secondary traumatic stress experienced by medical staff by witnessing the suffering of patients (22). Compassion fatigue originates from the “cost of caring” of frontline medical staff to patients suffering from psychological distress caused by COVID-19 (23). Medical staff during the COVID-19 outbreak worried about transmitting the virus to their families and friends albeit unintentionally (24). Such prolonged stress is highly likely to cause compassion fatigue. Compassion fatigue is a state of physical, emotional, social, and spiritual exhaustion of medical staff, which is caused by stress associated with prolonged contact with COVID-19 patients and intense fear of infection (25). Furthermore, health care workers suffering from compassion fatigue may be afraid of patients they care for, causing them to show avoidance behaviors in the doctor-patient relationship (26), which could be a way for them to cope with tremendous pressure. Many studies report that medical staff are normally full of compassion, which is an important quality required to provide patients with high-quality medical care (27). In general, medical staff can convert perceived stress into motivation to help patients, which encourages them to overcome the difficulties of their profession. However, in the case of prolonged work-related stress, they experience energy depletion and exhaustion, which leads to feelings of powerlessness, negative and intrusive thoughts, increased mental distance from the profession and patients, and eventually emotional fatigue (28). Based on the conservation of resources theory proposed by Hobfoll (29), individuals with sufficient resources to cope with demands from the internal and external environments are under less pressure. An individual can derive these resources intrinsically or extrinsically. Frontline medical staff are required to display great compassion when caring for COVID-19 patients. However, they may be unable to obtain sufficient resources to meet this demand due to the medical environment and pressure at the time. In this manner, their internal resources can be exhausted and cause symptoms of compassion fatigue, such as burnout and trauma. The fact that the symptoms of compassion fatigue and PSTD overlap indicates that compassion fatigue can aggravate PTSD symptoms. Thus, this study proposes the following hypothesis and the specific model hypothesis is shown in Figure 1.

Hypothesis 3: Compassion fatigue plays a moderating role in the relationship between perceived stress, insomnia severity, and PTSD symptoms.

## Methods

### Participants and Procedures

This study was conducted in August 2020. The researchers recruited medical staff from a northeast province, who provided support in Wuhan, Hubei Province during the lockdown due to the COVID-19 outbreak. The inclusion criteria were as follows: (1) the frontline medical staff were assigned to local hospitals in Wuhan to assist in epidemic control and prevention during the early stages of the COVID-19 outbreak. (2) The frontline medical staff provided their services for at least one and a half months. The exclusion criteria were as follows: (1) participants with incomplete questionnaires and (2) frontline medical staff restricted by their physical health to complete the questionnaire. A total of 1,209 frontline medical staff in a province in the northeast of China supported Wuhan in the fight against COVID-19. Among them, 659 frontline medical staff did not participate in the investigation due to work reasons, physical health, and failure to meet the inclusion criteria during the investigation. Thus, the conservative response rate was estimated to be 45.5%. After considering health and safety, the study used electronic questionnaires, which were distributed via a popular social network smartphone application called WeChat. Finally, a total of 550 questionnaires were collected for the study. After screening, a total of 516 questionnaires were returned for a valid response rate of 93.8%. Before data collection, the participants provided written informed consent and verbally confirmed the consent to the researchers.

### Instruments

#### PTSD Checklist-Civilian Version

The PCL-C is a widely used checklist to assess the PTSD status of patients after traumatic events and is used to measure the level of PTSD in medical staff (30). The PCL-C is composed of 17 items, which are rated using a five-point Likert-type scale to assess three symptom clusters, namely, re-experience, avoidance/numbing, and hyper-arousal. The total scores range from 17 to 85 (31). The higher the score, the more severe the PTSD symptoms. Moreover, participants who scored 38 or above are considered to have PTSD (32). Previous studies on emergencies reported that the scale displayed good reliability and validity (33). In the current study, the Cronbach's alpha coefficient for PTSD was 0.909.

#### Perceived Stress Scale

The Perceived Stress Scale (PSS) is composed of 10 items that evaluate the perceived stress of participants over the past month (34). Each item is rated using a five-point Likert-type scale (0 = never; 1 = almost never; 2 = sometimes; 3 = fairly often; 4 = very often). The total scores range from 0 to 40 points. The higher the score, the higher the level of perceived stress. Other studies widely used the scale, which displayed good reliability and validity (35). In the current study, Cronbach's alpha coefficient for the PSS was 0.712.

#### Insomnia Severity Index

The Insomnia Severity Index (ISI) is mainly used to assess the severity of subjective insomnia of participants in the past two weeks (36). The scale is composed of seven items, which are rated using a five-point Likert-type scale (0 = no problem; 4 = very severe problem). The total scores range from 0 to 28 points and are categorized into no insomnia (0–7), subthreshold (8–14), moderate (15–21), and severe (22–28) forms of insomnia. The scale displayed high reliability and validity in other studies (37, 38). The study established Cronbach's alpha at 0.923 as the reliability coefficient of the scale.

#### Compassion Fatigue Short Scale

Furthermore, the study employed Compassion Fatigue Short Scale (CFSS) developed by Adams, Boscarino, and Figley to evaluate compassion fatigue (39). The scale includes two dimensions, namely, (1) a five-item secondary trauma scale, and (2) an eight-item job burnout scale. The participants selected appropriate options based on their true feelings during the COVID-19 epidemic and rated each item using a 10-point Likert scale ranging from 1 (rarely/never) to 10 (very often) (40). The scale has been widely applied to Chinese emergency workers and displayed excellent constructs and cross-validation (40). Other studies in China reported the good reliability and validity of the scale and the excellent reliability of the subscales. The total scale was constructed in accordance with the original CF-Short Scale (39, 41). The current study found a Cronbach's alpha coefficient of 0.896.

### Data Analysis

Descriptive analysis was used for the basic sociodemographic characteristics of the participants, whereas correlation analysis was used to examine the associations among research variables. SPSS 24.0 (IBM Crop) and a PROCESS 3.2 macro were used to analyze the research variables. Statistical significance was set to *P* < 0.05. Moreover, the study employed one-way ANOVA to analyze differences between sociodemographic variables in PTSD. Multiple linear regression and the PROCESS macro were used to verify the mediating effect of insomnia severity and the moderating effect of compassion fatigue on all paths of the model. Finally, the study used 95% bootstrap confidence intervals (95% CI) based on 5,000 bootstrapped samples.

## Results

### Sample Characteristic

The study surveyed 516 medical staff who provide support in Wuhan to contain the spread of COVID-19. The age ranged from 21 to 65 years (mean = 37.74 years, SD = 8.87). The sample was composed of 415 (80.42%) women and 101 (19.58%) men, out of which 130, 349, and 37 participants had obtained a bellow university degree, university degree, and master's degree or above, respectively. Furthermore, 119, 376, and 21 participants were single, married, and divorced/widowed, respectively. One-way ANOVA found a statistical significance between gender and between those who were or were not worried about exposure to patients without symptoms. Table 1 provides the descriptive statistics of the participants.

**Table 1:** Demographic characteristics of the participants and associations with PTSD (*n* = 516).

**Variable**		***N* (%)**	**PTSD**	***F/t***
Gender				1.46^*^
	Female	415 (80.42)	26.08 ± 9.08	
	Male	101 (19.58)	27.38 ± 8.24	
Age				0.95
	21–35	249 (48.26)	26.67 ± 8.50	
	36–50	212 (41.09)	28.06 ± 8.78	
	51–65	55 (10.65)	25.56 ± 5.95	
Education				1.25
	Below University degree	130 (25.19)	27.09 ± 8.38	
	University degree	349 (67.64)	27.15 ± 8.55	
	Master's degree or above	37 (7.17)	26.97 ± 7.46	
Marital status				0.94
	Single	119 (23.06)	26.07 ± 7.92	
	Married	376 (72.87)	27.32 ± 8.53	
	Divorced/widowed	21 (4.07)	29.67 ± 8.75	
Employee type				1.28
	Nurse	328 (63.57)	27.62 ± 8.59	
	Doctor	101 (19.57)	26.94 ± 9.43	
	Medical technician	62 (12.02)	25.13 ± 5.32	
	Other	25 (4.84)	26.40 ± 7.64	
Technical title				0.80
	Other	16 (3.10)	28.50 ± 9.23	
	Junior	231 (44.77)	27.02 ± 8.37	
	Intermediate	153 (29.65)	26.77 ± 7.87	
	Senior	116(22.48)	27.62 ± 9.16	
Daily working hours during the epidemic				1.22
	<9 h	222 (43.02)	26.19 ± 7.76	
	9–10 h	101 (19.57)	27.31 ± 7.47	
	11–12 h	40 (7.75)	29.00 ± 9.88	
	>12 h	153 (29.66)	27.88 ± 9.39	
Are you worried about being exposed to asymptomatic infections				1.73^**^
	Yes	354 (68.6)	28.18 ± 8.70	
	No	162 (31.4)	24.83 ± 7.29	

* *P < 0.05;*

** *P < 0.001*.

### Preliminary Analyses

Table 2 displays the means, standard deviations, and bivariate correlations between research variables, which were positively correlated and exhibited significant statistical significance.

**Table 2:** Descriptive statistics and correlation among variables.

**Variables**	**1**	**2**	**3**	**4**
PTSD	1			
PS	0.46^**^	1		
ISI	0.69^**^	0.48^**^	1	
CF	0.62^**^	0.45^**^	0.51^**^	1
M	27.13	17.76	8.18	30.96
SD	8.42	5.23	5.87	17.49

*PS, perceived stress; ISI, insomnia severity index; CF, compassion fatigue*.

** *P < 0.01*.

### Testing for the Mediation Effect

This study employed multiple linear regression as proposed by Baron (42) to verify the mediation model of the study. As a result, the study constructed three models to verify the mediating effect of insomnia severity on the relationship between perceived stress and PTSD. Model 1 indicated that perceived stress had a significant predictive effect on PTSD (β = 0.458, *P* < 0.001), whereas model 2 pointed to the significant predictive effect of perceived stress on insomnia severity (β = 0.482, *P* < 0.001). Finally, when perceived stress and insomnia severity were included into the regression model as predictors, the study found that the predictive effect of PTSD remained significant. Furthermore, the study used SPSS-PROCESS macro (model 4) to further test the mediation model, which is based on the bootstrap method. The result indicated that 95% CI does not contain 0 [95% CI = (0.376, 0.583)], which indicates that perceived stress is not only related to PTSD but also indirectly related to PTSD through insomnia severity. For more information, see Table 3.

**Table 3:** Mediated regression analysis for PS and ISI on PTSD.

**Variable**	**Model 1** **(PTSD)**		**Model 2** **(ISI)**		**Model 3** **(PTSD)**	
	**β**	***t***	**β**	***t***	**β**	***t***
PS	0.458	11.685^***^	0.482	12.458^***^	0.163	4.564^***^
ISI					0.613	17.190^***^
*R^2^*	0.210		0.232		0.499	
*F*	136.541^***^		155.216^***^		255.143^***^	

*PS, perceived stress; ISI, insomnia severity index; CF, compassion fatigue*.

*** *P < 0.001*.

### Testing for the Moderated Mediation Effect

As displayed in Table 4, perceived stress (β = 0.274, *P* < 0.001) but not compassion fatigue (β = 0.045, *P* > 0.05) can significantly predict insomnia. However, the interaction terms of perceived stress and compassion fatigue (β = 0.003, *P* > 0.05) remained non-significant in predicting insomnia severity.

**Table 4:** Testing the moderated mediation effect of CF.

**Variable**	**β**	**SE**	***t***
Mediator variable model (Outcome: ISI)			
PS	0.274	0.068	3.976^***^
CF	0.045	0.048	0.944
PS × CF	0.003	0.002	1.647
Dependent variable model (Outcome: PTSD)			
PS	−0.132	0.096	−1.365
ISI	0.577	0.094	6.098^***^
CF	−0.103	0.054	−1.890
PS × CF	0.011	0.003	3.559^***^
ISI × CF	0.003	0.002	1.185

*PS, perceived stress; ISI, insomnia severity index; CF, compassion fatigue*.

*** *P < 0.001*.

Thus, compassion fatigue does not moderate the relationship between perceived stress and insomnia severity. In the next step, the study used PTSD as the dependent variable to verify whether compassion fatigue plays a role in moderating the relationship between perceived stress and PTSD as well as between insomnia severity and PTSD. The results indicated that the interaction between perceived stress and compassion fatigue could be significant in predicting PTSD (β = 0.011, *P* < 0.001). In other words, compassion fatigue moderated the direct effects of the moderated mediation. However, the interaction between insomnia severity and compassion fatigue in predicting PTSD was statistically non-significant (β = 0.003, *P* > 0.05). Thus, compassion fatigue does not exert a moderating effect on the relationship between insomnia severity and PTSD.

**Figure 2:**
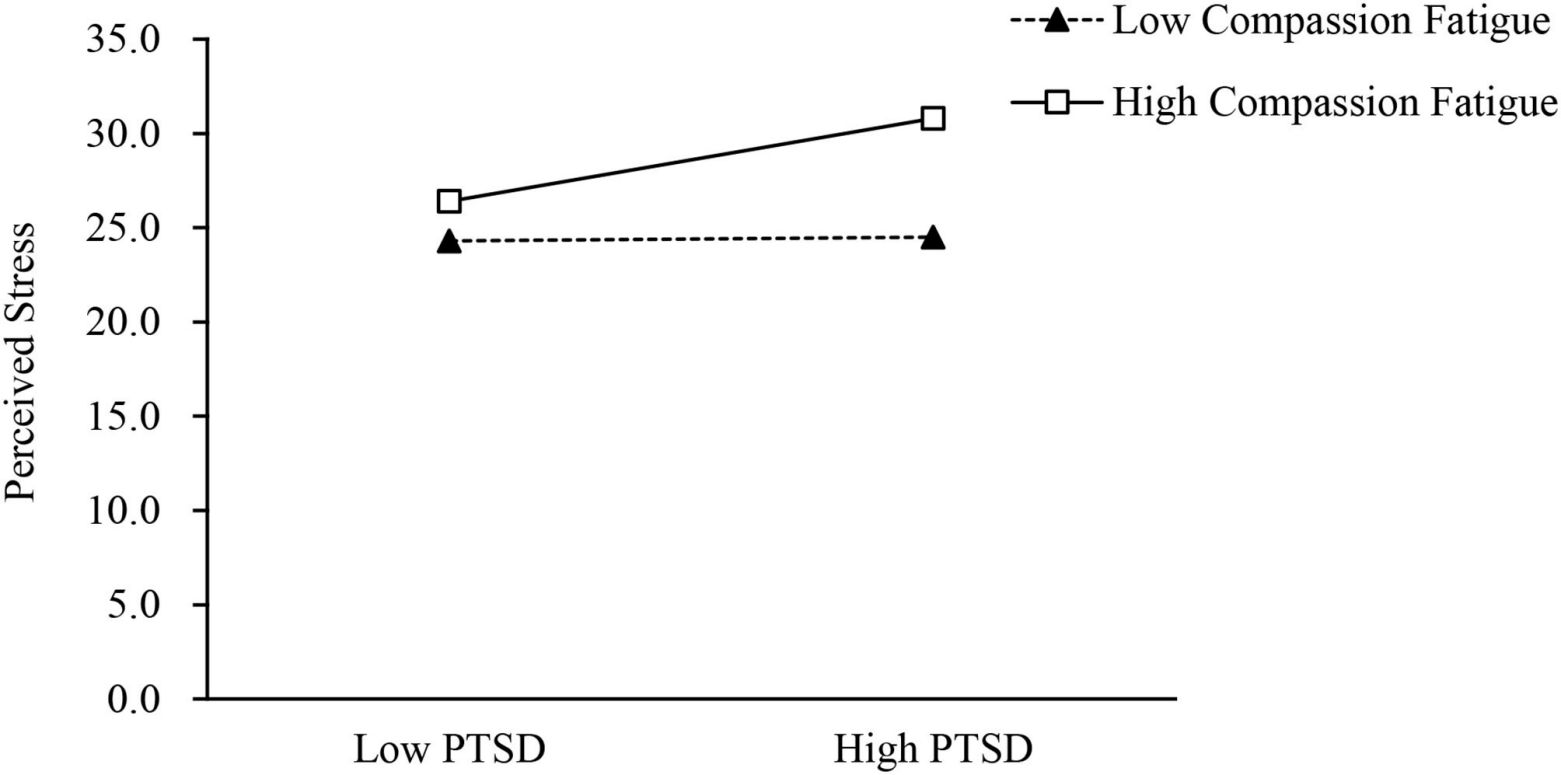
Compassion fatigue moderates the effect of the perceived stress on PTSD.

To further verify the moderated mediation model, the study applied the PROCESS macro method (model 59), which is based on the bootstrap method. The result indicated that compassion fatigue moderates the relationship between perceived stress and PTSD because the 95% CI does not contain 0 [95% CI = (0.005, 0.018)]. However, compassion fatigue did not moderate the relationship between perceived stress and insomnia severity [95% CI = (−0.001, 0.008)] and between insomnia severity and PTSD [95% CI = (–0.002, 0.008)]. To further illustrate the moderating effect of compassion fatigue, the study used a simple slope test. As shown in Table 5 and Figure 2, high levels of perceived stress were associated with high levels of PTSD (β_simple_ = 0.415, *t* = 4.562, *P* < 0.001) among individuals with high levels of compassion fatigue. However, for individuals with low levels of compassion fatigue, the moderation effect was non-significant (β_simple_ = 0.020, *t* = 0.306, *P* > 0.05).

**Table 5:** The moderating effect of compassion fatigue.

**Variables**		**Effect**	**Boot 95% CI**
Conditional indirect effect analysis			
1 SD below the mean	13.471	0.020	(−0.108, 0.149)
Mean	30.957	0.217	(0.106, 0.329)
1 SD above the mean	48.444	0.415	(0.236, 0.594)

## Discussion

The study aimed to explore the potential connections between PTSD, perceived stress, insomnia severity, and compassion fatigue among medical staff from the northeast province who provided medical support in Wuhan during the lockdown due to the COVID-19 outbreak. The study found that 10.5% of the medical staff were reported with PTSD symptoms. This result is in close agreement with those found for rapid systematic reviews and meta-analyses on the reported rates of PTSD among medical staff during the COVID-19 pandemic (43). However, this rate for PTSD was higher than those of the Chinese public (4.6%) one month after the COVID-19 outbreak in China (44) and a similar report on PTSD (10.4%) among Chinese adolescents in June 2020 (45). This comparison highlighted the importance of paying attention to the PTSD status quo of frontline medical workers in Wuhan during the fight against COVID-19. Furthermore, the current study was conducted in August 2020. In terms of the timeframe, relevant studies illustrated that PTSD displayed a downward trend over time after traumatic events (46). However, the current study found that after 6 months of supporting Wuhan, the levels of PTSD symptoms of the medical staff remained relatively high. Therefore, even during the normalization phase of the epidemic, offering and implementing psychological interventions for medical staff with PTSD symptoms are considered very important. In addition, the study found a statistical significance between gender differences and whether the participants were concerned about exposure to asymptomatic infection and PTSD symptoms. The results suggested that men were more likely to exhibited PTSD symptoms than women, which is consistent with the results of a study conducted during the COVID-19 epidemic (8), although contrary to the results of other studies on COVID-19 (47). The differences could be the result of differences in the times when these studies were conducted. Female medical staff may display PTSD symptoms when they first participated in the fight against COVID-19, which disappeared over time (48). In addition, female medical staff also tended to pay more attention to their own experiences and feelings, and are more willing to express their emotions with family and friends, which is conducive to for the self-regulation of emotions (49). However, male medical staff may opt out of self-emotion regulation, such that they recover more slowly than women and show more PTSD symptoms. Similarly, medical staff who were concerned about asymptomatic infection are more likely to exhibit PTSD symptoms. A possible reason for this finding is that during the COVID-19 outbreak, medical staff worked in high-risk environments, which led to concerns about potential health hazards (7), and, thus, asymptomatic infection. These results indicated that increased attention should be paid to the PTSD symptoms displayed by medical staff and that effective measures should be taken to alleviate these symptoms in the future.

As expected, insomnia severity mediated the effect of perceived stress on PTSD, which confirmed the above hypothesis. In the process of supporting Wuhan against COVID-19, the medical staff faced tremendous pressure in the form of shortage of medical supplies and stress as a result of the lack of effective treatment of the disease at the time (50, 51). During the COVID-19 epidemic, workplace stress for medical staff is an important factor that should be considered as well as other problems, such as lack of sleep, because perceived stress is a risk factor for the development of sleep issues among frontline medical staff (52, 53). This suggestion applies to the concept of allostasis stress proposed by Sterling and Eye (54). If an individual is under extreme stress for a prolonged period of time, then an allostasis state of mind and body occurs, which is manifested in various forms, such as sleep disorders and excessive emotional arousal. Previous studies reported that COVID-19 reduced the sleep quality of individuals during the epidemic (55), which increases the risk of infection among frontline medical staff due to direct contact with patients with COVID-19 (56). Under such circumstances, frontline medical staff with perceived high levels of stress may experience sleep disorders. In turn, medical staff with poor sleep quality are prone to PTSD-related symptoms (57). Furthermore, sleep problems, such as insomnia and nightmares, are considered common symptoms of PTSD, which suggests that medical staff with poor sleep quality are more vulnerable to PTSD symptoms (58). Thus, the current study proposes that sleep-related problems mediate the relationship between perceived stress and PTSD among frontline medical staff. Relevant studies demonstrated that good sleep quality plays an important role in restoring neurobehavioral function and in alleviating psychological distress, such as depression, stress, and PTSD (59). Therefore, relevant agencies should reset work schedules, promote frequent breaks during work shifts (60), and establish a shift work model that respects the health and wellbeing of medical staff (61). These measures can provide medical staff with a good sleeping environment to enhance immunity, which may reduce stress and PTSD.

The results indicated that compassion fatigue only moderates the association between perceived stress and PTSD. This moderating effect is very significant among medical staff with high levels of compassion fatigue, whereas no moderating effect was observed for medical staff with low levels of compassion fatigue. Individuals with high levels of compassion fatigue will exhibit a state of physical and mental fatigue and reduced pleasure and satisfaction at work, which results in a general decline in energy and ability to help and rescue others (62, 63). Compassion fatigue mainly comes from secondary traumatization, which is different from primary traumatization. It emphasizes on the cumulative effect of stress caused by continuous expression of empathy of frontline medical staff toward COVID-19 patients (64). On the other hand, primary traumatization is defined as a stress response of an individual from directly experiencing a traumatic event. The effect is obvious in a high compassion fatigue state, but in a low level of compassion fatigue state, exhaustion can turn into compassionate satisfaction, the pleasure and satisfying feeling that comes from helping others. During the COVID-19 epidemic, frontline medical staff faced tremendous levels of stress, treated a large number of patients, lacked equipment, had to use unfamiliar equipment, and lacked specific evidence that could be used to guide disease treatment (65). Thus, they were exposed to the risk of developing compassion fatigue, which is conceptualized as a response to indirect exposure to traumatic events. Individuals who reported more severe PTSD symptoms also reported high levels of compassion fatigue (66). According to the compassion fatigue model (CFM), everyone has their own balance of resources. Frontline medics always facing tremendous pressure from their working environments, if they lack resources to cope with the pressure, they will experience severe personal distress. Once the balance of resources is broken, medical staff will suffer from compassion fatigue and show symptoms of PTSD (67). However, this study did not observe the moderating effect of low levels of compassion fatigue. The reason for this result may be that previous studies observed high levels of compassion in a large proportion of frontline medical staff who participated in the rescue. One study suggested that when medical staff believed in their causes, such as rescuing patients with COVID-19 and saving lives, such positive beliefs and thoughts may have provided them with a sense of accomplishment and satisfaction, which, in turn, eased their fears and provided protection from stressful events that may result in PTSD symptoms (68). Thus, the goal of compassion fatigue research is to help caregivers to build strong psychological resilience to enrich their internal resources, so they can quickly recover from traumatic experiences and maintain efficient and high function work performances (62). Effective strategies to prevent and control compassion fatigue are through internal self-awareness and efficient self-care and management. Health managers should encourage frontline medical staff to recover from compassion fatigue by improve communication skills, self-expression, and self-compassion (69).

The study has several limitations. First, this study was based on a cross-sectional survey. Thus, pinpointing the causal relationships between research variables is impossible. Furthermore, the research was based on a questionnaire. Therefore, the results may be prone to subjectivity and reliability. Lastly, the study only explores the relationship between insomnia severity and compassion fatigue between perceived stress and PTSD. Thus, future research should explore the relationship between other variables.

Despite these limitations, the current study shed light on the relationship between perceived stress and PTSD and emphasized that PTSD among frontline medical workers in the fight against the COVID-19 epidemic largely remains a concern. Moreover, the study confirmed that perceived stress is not only directly related to PTSD but also indirectly related to PTSD through insomnia severity with the moderating role of compassion fatigue on the direct path. Therefore, the study provided reference for the formulation of psychological intervention programs for frontline medical staff affected by PTSD during other similar public health emergencies.

## Data Availability Statement

The raw data supporting the conclusions of this article will be made available by the authors, without undue reservation.

## Author Contributions

SM, YCH, and LL conceived the presented idea. HR, YYH, ZQ, RC, CL, JF, TY, CM, XG, and JL discussed the results and contributed to the final manuscript. All authors contributed to the article and approved the submitted version.

## Conflict of Interest

The authors declare that the research was conducted in the absence of any commercial or financial relationships that could be construed as a potential conflict of interest.

## Publisher's Note

All claims expressed in this article are solely those of the authors and do not necessarily represent those of their affiliated organizations, or those of the publisher, the editors and the reviewers. Any product that may be evaluated in this article, or claim that may be made by its manufacturer, is not guaranteed or endorsed by the publisher.
